# Neoadjuvant Endocrine Therapy Compared to Neoadjuvant Chemotherapy in Node-Positive HR+, HER2− Breast Cancer (Nodal pCR and the Rate of ALND): A Systematic Review and Meta-Analysis

**DOI:** 10.1155/2024/8866756

**Published:** 2024-08-02

**Authors:** Mahtab Vasigh, Mohammadreza Karoobi, Austin D. Williams, Fasika Molla Abreha, Richard J. Bleicher, Seyed Mostafa Meshkati Yazd, Tahereh Shamsi, Ramesh Omranipour, Ahmad Elahi, David Farhat, Mehran Habibi

**Affiliations:** ^1^ Department of Surgical Oncology Fox Chase Cancer Center, Philadelphia, PA, USA; ^2^ Department of Surgery Tehran University of Medical Sciences, Tehran, Iran; ^3^ Breast Disease Research Center Tehran University of Medical Sciences, Tehran, Iran; ^4^ Department of Surgery Johns Hopkins Medical Institute, Baltimore, MD, USA; ^5^ Department of Surgery Alborz University of Medical Sciences, Alborz, Iran; ^6^ Donald and Barbara Zucker School of Medicine Hofstra/Northwell University, Hempstead, NY, USA

## Abstract

**Introduction:**

Patients with hormone receptor-positive (HR+), HER2-negative (HER2−) breast cancers have the lowest response to neoadjuvant therapy of all subtypes. The role of neoadjuvant endocrine therapy (NET) in clinically node-positive (cN+), HR+, HER2− patients is evaluated in this meta-analysis.

**Methods:**

This study was performed between January 2010 and August 2022. We evaluated the node pathologic complete response (pCR) and axillary lymph node dissection (ALND) rates after neoadjuvant endocrine therapy (NET).

**Results:**

18,037 HR+, HER2−, cN+ stage II and stage III breast cancer patients within eleven studies received neoadjuvant treatments. 3,707 (20.6%) patients received NET and 14,330 (79.4%) received NAC. The average age of the NET patients was higher than that of the neoadjuvant chemotherapy (NAC) patients (64.1 versus 47.6 years old, *p* < 0.001). 45.0% and 26.9% of the NET and the NAC groups underwent a lumpectomy. The pooled estimates of node pCR in NET and NAC groups were 8.9% and 14.9%, and the pooled proportion of ALND was 39.1% and 58.5%, respectively.

**Conclusion:**

The rate of node pCR was lower among cN+ patients who received NET compared to the NAC group. The rate of ALND among cN+ NET patients was lower than the NAC group, revealing more patients with residual nodal disease do not get ALND in the NET group. Further prospective studies are required to compare survival outcomes as a more reliable surrogate.

## 1. Introduction

Breast cancer is the most common cancer among women in the United States [1], and hormone receptor-positive (HR+), human epidermal growth factor receptor 2 negative (HER2−) is the most common subtype [2–4]. Neoadjuvant therapy (NT) for breast cancer is a well-established therapeutic approach in certain high-risk patients, including those with large tumors or positive lymph nodes. NT can decrease tumor size in large tumors, making some patients eligible for breast-conserving surgery or downstage locally advanced and unresectable primary breast cancers to facilitate resection [5, 6]. NT can also eradicate nodal disease, allowing for the avoidance of axillary lymph node dissection (ALND) and its associated morbidity in patients who achieve a pathologic complete response (pCR) in the lymph nodes [7–9]. Recently, researchers have shown a growing interest in studying long-term survival outcomes for clinically node positive (cN+) patients who achieve pathologic negative nodes after neoadjuvant chemotherapy (ypN0) status after neoadjuvant chemotherapy (NAC), resulting in the emergence of a fresh and developing body of literature on this subject [10]. Notably, the five-year oncologic outcomes of cN+ patients who become ypN0 following NAC are similar to those in the American College of Surgeons Oncology Group (ACOSOG) Z11 [11] and AMAROS [12] groups with a positive sentinel lymph node biopsy (SLNB) without further ALND [13]. Failure to achieve node pCR after NAC is a poor prognostic sign, as evidenced by the significant reduction in 5-year disease-free survival (DFS) and overall survival (OS) in the ALND group with further positive disease [13–15]. However, further data are needed to determine if this also applies to patients undergoing neoadjuvant endocrine therapy (NET) and to assess the safety of omitting ALND in patients who fail to achieve nodal pCR after NET.

It is well documented that patients with HR+ breast cancer are less likely than those with HER2+ or triple-negative breast cancer to achieve a pCR after treatment with neoadjuvant chemotherapy (NAC) [7, 16, 17]. Outcomes from the I‐SPY trial demonstrate that patient outcomes can substantially differ based on molecular tumor markers; in the trial, the majority of patients were clinical N1/pathologic N1 (cN1/pN1), and HR+, HER2− patients experienced the lowest rate of pCR after NAC (9% for HR+, 33% for HR+, HER2+, 35% for triple-negative, and 58% for HR−, HER2+) [18]. ACOSOG's Z1071 results have similar findings of significantly lower rates of node pCR (21%) after NAC in HR+, HER2− tumors compared with HER2+ or triple-negative tumors [19].

Due to this inadequate response to NAC, neoadjuvant endocrine therapy (NET) has become an exciting alternative for hormone-receptor positive (HR+) breast cancer patients. Primary endocrine therapy was initially used for elderly patients, ineligible for systemic chemotherapy or upfront surgery, given its limited toxicity [20–22]. Application to the neoadjuvant setting became more common during the COVID-19 pandemic [23] and has shown promise with breast conservation rates comparable to those of NAC [24]. However, a nodal pCR is currently required to avoid ALND among patients with cN+ disease [25]. In light of the lower pCR rate, NET may not accomplish the goal of minimizing surgical morbidity [26, 27]. Considering the lower toxicity of NET compared to NAC and the increased use of NET during the pandemic [28], we performed a systematic review and meta-analysis to evaluate the role of NET in cN+, HR+, HER− patients.

## 2. Methods

### 2.1. Search Strategy

This systematic review followed the Preferred Reporting Items for Systematic Reviews and Meta-Analyses (PRISMA) guidelines. We searched databases including Scopus, Embase, PubMed, and the Web of Science from January 2010 to August 2022 using the following keywords: (“locally advanced breast cancer” [MeSH Terms] OR “locally advanced breast cancer” [All Fields]) AND “node-positive” [All Fields] AND (“hormone receptor-positive” [All Fields] OR “hormone receptor-positive” [MeSH Terms]) AND “HER2 negative” [All Fields] AND (“neoadjuvant endocrine therapy” [All Fields] OR “neoadjuvant endocrine therapy” [MeSH Terms]). Inclusion and exclusion criteria were based on the population‐intervention‐comparator‐outcomes study design (PICOS) framework. Included were prospective and retrospective studies involving neoadjuvant endocrine therapy that reported axillary pathologic response in clinically node+, HR+, HER2− patients. This study was approved by the review board at Tehran University of Medical Sciences.

### 2.2. Study Selection

**Figure 1:**
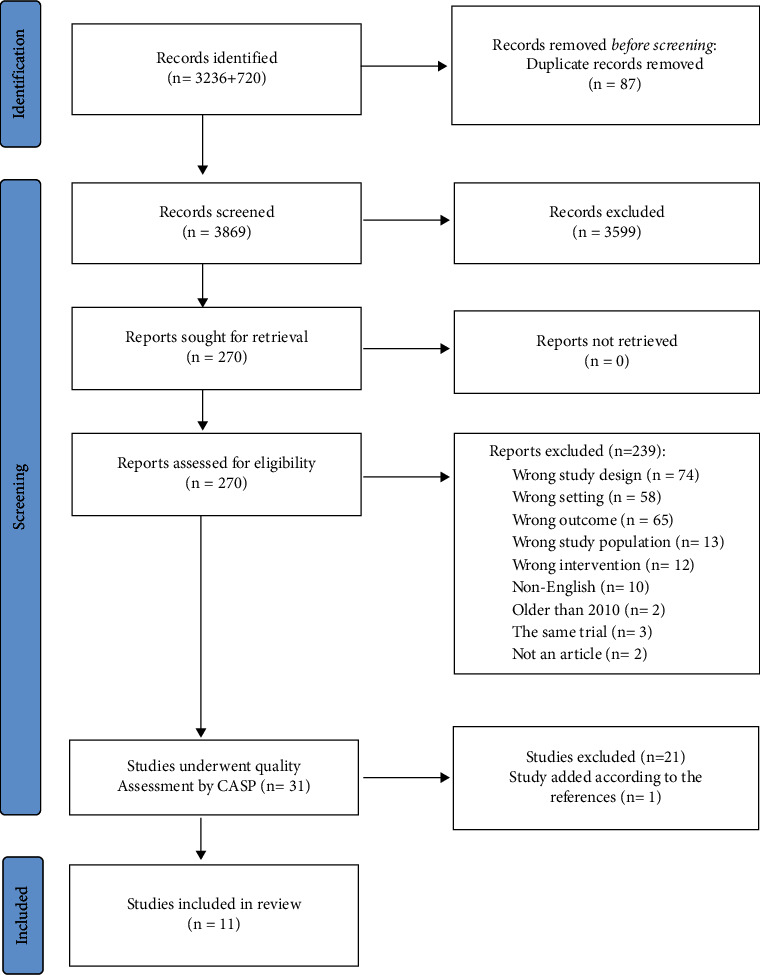
PRISMA flow diagram for search results and selection details.

We identified 3,956 potentially relevant citations and entered them into the Covidence systematic review management system. Covidence removed 87 duplicate citations. Two independent reviewers (MK and TS) eliminated 3,599 citations based on irrelevance, including conference abstracts, reviews, editorials, letters, comments, and irrelevant abstracts. Two reviewers (MV and AE) then assessed the full texts of 270 papers, excluding 239 publications for the following various reasons: wrong design (*n* = 74), wrong setting (*n* = 58), wrong outcomes (*n* = 65), wrong population (*n* = 13), wrong intervention (*n* = 12), non-English language (*n* = 10), published before 2010 (*n* = 2), studies from the same trial (*n* = 3), and poster-only presentations (*n* = 2). The quality of the remaining studies was evaluated using the Critical Appraisal Skills Programme (CASP) checklist. The PRISMA flowchart in Figure 1 illustrates the literature search and study selection process.

### 2.3. Outcomes: Data Extraction

We evaluated two endpoints in 11 studies [29–39]: node pCR and the rate of ALND. Overall survival (OS) was reported in only four studies; due to heterogeneity, a meta-analysis on OS was not feasible. Data were extracted by two authors (MV and MM) and reviewed by a third investigator (MK) for accuracy. We did not contact the original study investigators for raw data. A predesigned form captured basic study features, design, study population (clinical tumor stage, histologic subtype, tumor grade, duration of NET, and age), outcomes, and related proportions (node pCR and ALND). We extracted the number of incidents and total sample size directly from the studies; when only percentages were reported, rounding was used to estimate incident numbers.

### 2.4. Statistical Analysis

Statistical analyses were performed using STATA (version 18; Stata Corp, College Station, Texas, USA). We assessed statistical heterogeneity using Higgin's *I*-square test and Cochrane's *Q* test, with *I*^2^ > 50% or *Q* test *p* values <0.05 indicating heterogeneity. Random effect analysis was used to neutralize heterogeneity (*I*^2^ > 50%). We visually assessed the likelihood of publication bias through funnel plots, Begg's and Egger's tests, and Duval and Tweedie's trim-and-fill method. Proportions reported in each study were compared using the Chi-square test.

The primary and secondary outcomes were node pathologic complete response and axillary dissection, respectively. We used the “metan” command in STATA for meta-analysis, computing summary estimates of variables of interest. We pooled the effect sizes using odds ratios (ORs) as all outcomes were binary variables.

## 3. Results

### 3.1. Publication Bias and Heterogeneity

**Figure 2:**
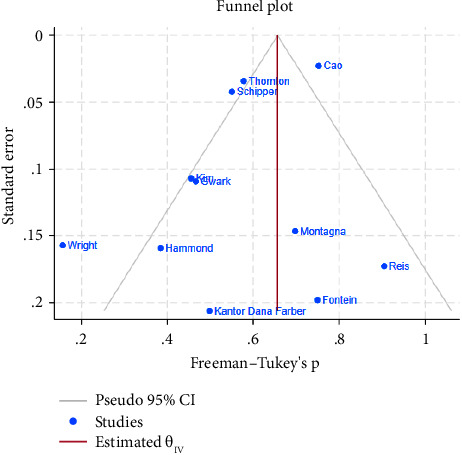
Funnel plot for assessment of publication bias.

**Figure 3:**
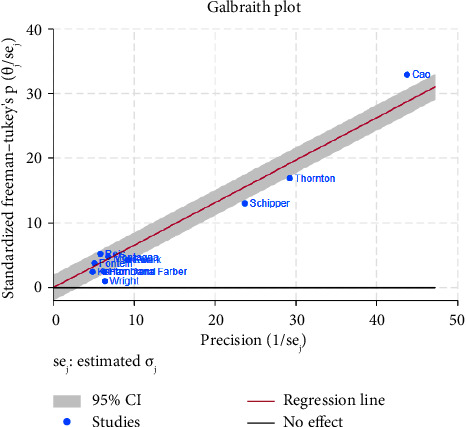
Galbraith plot for indicating the sources of heterogeneity among meta-analysis results.

The funnel plot for publication bias indicated minimal bias across all 11 studies (Figure 2). Kendall *τ* statistics yielded 0.78 (*p*: 0.4), suggesting no evidence of publication bias, and the Egger test confirmed this finding (*p*: 0.6; 95% CI: −3.04, 1.93). The *I*-square values above 50% in all forest plots indicated substantial heterogeneity, primarily driven by differences between studies rather than sampling variation. A Galbraith plot analysis identified sources of heterogeneity (Figure 3), with *I*-square decreasing to 74.7% (*p* value = 0.130) after outlier exclusion, while maintaining a stable overall estimate for node pCR.

### 3.2. Patient Characteristics and Treatment Comparison

In total, 27,569 HR+, HER2− stage II and III breast cancer patients were included from eleven studies (Table 1). Neoadjuvant endocrine therapy (NET) was administered in 7 out of 11 studies, with durations ranging from 16 to 34 weeks. A comparison between NET and neoadjuvant chemotherapy (NAC) across six studies showed that 7,842 out of 27,569 patients (28.4%) received NET (Table 1). The average age of patients in the NET group was 64.1 years, higher than the NAC group's average of 47.6 years. The majority of patients in both groups were Caucasian, with 84.6% (95% CI: 83.7%–85.5%) in the NET group and 80.6% (80.1%–81.2%) in the NAC group. Pathology findings indicated invasive ductal carcinoma (IDC) as the most common subtype in both groups (Table 2).

### 3.3. Pathologic Response Rates

Meta-analysis findings revealed a pCR in breast tissue of 2.2% (95% CI: 0.2–4.2) for the NET group and 6.7% (95% CI: 6.3–7.0) for the NAC group. Notably, lumpectomy rates were higher in the NET patients (45.0%; 95% CI: 43.8–46.1) compared to the NAC patients (26.9%; 95% CI: 26.3–27.5).

### 3.4. Node Pathologic Complete Response and Axillary Dissection

**Figure 4:**
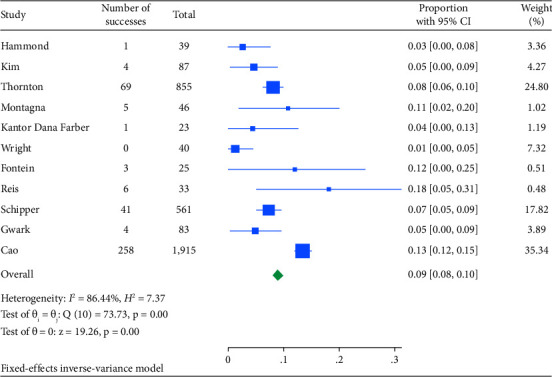
Forest plot for the pooled proportion of node PCR by NET.

**Figure 5:**
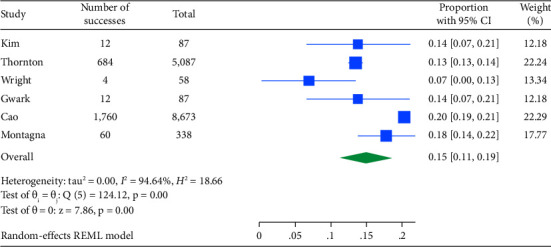
Forest plot for the pooled proportion of node PCR by NAC.

Among 18,037 clinically node-positive (cN+) patients, confirmed by histology in 1112 (6.1%), NET was administered to 47.2% and NAC to 72.6% (Table 3). Node pCR rates were 8.9% (95% CI: 8.0–9.8) for NET and 14.9% (95% CI: 11.2–18.7) for NAC (Table 3). Figures 4 and 5 illustrate these findings. The rate of axillary lymph node dissection (ALND) was lower in the NET group (39.1%) compared to the NAC group (58.5%) (Table 2). Within the NET group, 9.6% did not undergo ALND and were found to have isolated tumor cells or microscopic nodal disease, while 29.2% were undefined or pN1.

### 3.5. Survival Benefit Review

The mean follow-up period in different studies was reported to be between 29.7 and 60 months. Four large studies reported the OS or recurrence rates in NAC and NET patients [32, 35, 37, 38], but due to the heterogeneity of the studies, a meta-analysis on OS was not feasible. Thornton et al. selected cT1-4, LN+ (cN1-3), HR+, and ILC patients between 2004 and 2014 who received either NET or NAC from the National Cancer Database (NCDB). After adjustment for covariates, there was no significant difference in OS between patients who received NET and NAC in their study (HR 0.84, 95% CI 0.68–1.03, *p*=0.10) [38]. Similarly, Wright demonstrated equivalent outcomes in patients who received endocrine therapy only compared with those who received cytotoxic chemotherapy as a component of their regimen based on their response to NET. There were no apparent differences in locoregional recurrence (LRR), distant metastasis (DM), and overall recurrence (OR) among the groups in the study by Wright [39].

## 4. Discussion

### 4.1. Nodal Pathologic Complete Response

In this meta-analysis, we found that HR+, HER2−, cN+ breast cancer patients undergoing NET are less likely to achieve node pCR (8.9%) than patients receiving NAC (14.9%).

The node pCR (21.1%) in HR+, HER2− patients following NAC in the ACOSOG Z1071 trial was higher than our findings [19]. The node pCR rate for HR+, HER2− patients following NET in the ACOSOG Z1031 was lower than NAC (1.8% compared to 8%) [40]. Their node pCR rate for NET and NAC patients was lower than our results.

### 4.2. ALND

Our study showed that although the node pCR rate is higher after NAC compared to NET, NAC patients more frequently undergo ALND (58.5%) than NET patients (39.1%). Two variations in surgeons' approach to axillary management after NET may address this paradox. First, some patients with a small burden of residual nodal involvement after NET do not undergo ALND. Second, more patients with a node pCR after NAC still undergo ALND. For patients with cN+ disease undergoing NAC, SLNB may be performed in those who are cN0 after treatment, and ALND may be avoided if the patient achieves node pCR (ALND sparing) [41, 42] Several studies on axillary management after NAC are available [14], but more data on axillary management after NET are needed. Until the results of the Alliance A011202 trial [43] (which compares ALND+ axillary radiation to axillary radiation alone in patients with cN+ disease who fail to achieve a node pCR after NAC), the current standard for cN+ patients with residual nodal disease after NAC is ALND. [44, 45] The present study showed that despite the lower node pCR rate in NET, fewer patients in the NET group undergo ALND compared to NAC group. It indicates that more patients with residual nodal disease in the NET group do not undergo ALND. Our results are consistent with the findings of the ACOSOG Z1031 trial, which reported that despite similar rates of node positivity in the AI-alone (44%) and the NAC (43%) cohorts, patients who received AI alone were less likely to undergo ALND (33% vs. 69%) [40]. Another study evaluating the NCDB reported that fewer NET-treated patients with positive nodes go on to receive ALND compared to NAC-treated patients [45]. It was revealed in a study from the NCDB between 2010 and 2016 that patients receiving NAC and achieving node pCR were more likely to undergo an ALND than NET patients with node pCR (54.7% vs. 43.8%) [35, 37, 46].

### 4.3. Survival Benefit

Although the frequency of pCR in HR+ tumors is low, the strength of the association of pCR and OS is lower in HR+ subgroups [47]. Breast cancer survival is relatively higher, despite lower pCR following both NET and NAC, in HR+, HER2− patients compared to triple-negative or HER2+ subtypes, indicating more indolent tumor biology. Therefore, unlike the other subtypes of breast cancer, pCR after NET may not be as good a surrogate endpoint for long-term outcomes in patients with HR+, HER2− patients, even in those with cN+ disease [48, 49]. Due to heterogeneity in the existing studies, we could not perform a meta-analysis on OS after NET. However, the studies reporting this outcome indicate no difference in OS between those receiving NET and NAC.

Kantor et al. reported that small-volume residual nodal disease after NET, defined as isolated tumor cells or micrometastases, was not associated with worse survival outcomes than ypN0 patients [35, 46]. They hypothesized that leaving behind a low volume of axillary disease following NET is potentially less important than following NAC, as NET patients have only received a small fraction of their overall endocrine therapy in the preoperative setting [35, 46].

### 4.4. Strengths and Limitations

Our study-level meta-analysis has several strengths. We used a comprehensive, systematic, unbiased search strategy with relevant databases. This study provides insight into the efficacy of NET for cN+, HR+, HER2− breast cancer; however, it has several limitations. First, our study could analyze node pCR and ALND rates as efficacy measures, which are not likely the most important outcomes that should drive surgical decision-making; additional clinical trials evaluating the correlation between NET, axillary response, and survival outcomes are necessary to provide these answers. Second, this study is a study-level meta-analysis. Other than the possibility of errors in reporting, the lack of information can severely limit the type of analyses and conclusions. Third, the agents used in NAC and NET regimens were infrequently reported. Therefore, despite our best efforts to reduce cross-trial imbalances by examining subgroups and baseline patient characteristics for the target population, subpopulation imbalances persisted, resulting in an underestimation of cross-trial heterogeneity. Finally, some of the studies in this meta-analysis were retrospective, which may increase the selection bias.

## 5. Conclusions

This meta-analysis confirmed that node pCR is lower in HR+, HER2−, cN+ breast cancer patients following NET than NAC. Conversely, pooled estimate analysis also revealed a lower rate of ALND after NET compared to NAC, indicating that more patients with residual nodal disease following NET did not undergo ALND compared to NAC. While it is clear that surgeons' practice patterns differ when treating patients who have received NET or NAC, we are unable in the current study to determine whether these variations exist due to a lack of knowledge or agreement and their impact on LRR and OS. Further prospective trials are required to determine the influence of leaving behind residual nodal disease and not performing ALND on LRR and OS following NET.

## Figures and Tables

**Table 1:** Summary of the studies included in the meta-analysis.

	Author	Year	Study design	Total *n*	NET *n*	NET regimen	NET mean duration weeks	NAC regimen	NAC mean duration (weeks)	Primary endpoint	Secondary endpoint
1	Fontein [28]	2014	RCT	102	102	Exemestane	25			Imaging clinical response	BCS/pCR
2	Wright [33]	2015	Retrospective cohort	140	57	AI or tamoxifen	34	Platinum, anthracycline, and taxane containing 44 (31.4%) TC: 38 (27.1%) Unknown 1 (0.7)		pCR, recurrence rate	LRR
3	Thornton [32]	2019	Cohort	5942	855	Unknown	Unknown	Unknown	Unknown	OS	
4	Montagna [30]	2020	Cohort	465	127	Unknown	20	ddACT: 95% CMF: 4% TC: 1%		pCR	Downstaging
5	Hammond [25]	2020	Retrospective cohort	42	39	AI + leuprolide	18			SLNB/pCR	Final nodal staging
6	Kim [27]	2020	RCT	174	87	Goserelin and tamoxifen	24	ACT	8 cycles	MRI response	BCS, Ki67
7	Kantor [29] DFBWCC	2020	Cohort	94	94	Unknown	17			OS/SLNB	Residual nodal RF
8	Reis [23]	2022	Prospective cohort	33	33	Letrozole + exemestane	16			MRI characteristics	pCR
9	Schipper [24]	2021	Retrospective cohort	561	561	AI or tamoxifen ± GnRH agonist	34			pCR	pCR factors
10	Cao [31]	2021	Retrospective cohort	19829	5804	Unknown	Unknown	Unknown	Unknown	pCR/SLNB	OS
11	Gwark [26]	2021	RCT	187	83	Goserelin and tamoxifen	24	ACT	8 cycles every three weeks	SLNB	DFS/OS
Total				27,569	7,842						

DFBWCC: Dana-Farber/Brigham and Women's Cancer Center, RCT: randomized controlled trial, SLNB: sentinel lymph node biopsy, OS: overall survival, pCR: pathologic complete response, BCS: breast-conserving surgery, DFS: disease-free survival, AI: aromatase inhibitor, LRR: locoregional recurrence. ddACT: dose-dense anthracycline and taxane-based chemotherapy, CMF: cyclophosphamide, methotrexate, and 5-fluorouracil, TC: docetaxel and cyclophosphamide.

**Table 2:** Pooled estimate prevalence (%) of clinicopathologic characteristics of HR+, HER2- patients who received neoadjuvant treatment with 95% CI (upper limit-lower limit).

Variable	NET (*n* = 14484)	NAC (*n* = 30721)
	Pooled proportion % with (95% CI)	Pooled proportion % with (95% CI)
Age (mean/years)	64.1	47.6
Race		
White	84.6 (83.7–85.5)	80.6 (80.1–81.2)
Black	10.7 (9.9–11.4)	14.1 (13.6–14.6)
Hispanic	4.9 (3.5–6.2)	6.6 (6.0–7.3)
Other or unknown	4.0 (3.6–4.5)	4.3 (0.4–4.6)
Histology		
IDC	59.6 (42.4–76.8)	61.8 (29.0–94.6)
ILC	34.0 (16.5–51.6)	29.5 (0–64.4)
IDC and ILC	10.3 (2.4–18.2)	—
Other or unknown	3.8 (1.4–6.3)	2.4 (0.5–4.3)
Clinical *T* stage		
cT1	26.5 (25.5–27.5)	11.7 (10.9–12.5)
cT2	35.4 (34.3–36.4)	47.6 (46.9–48.3)
cT3	20.2 (19.3–21.1)	28.8 (28.2–29.4)
cT4	9.5 (8.9–10.2)	15.1 (14.6–15.6)
Pathologic *T* stage		
pT0	0.6 (0.4–0.8)	3.6 (3.1–4.1)
pT1	23.7 (22.7–24.7)	23.1 (21.9–24.2)
pT2	46.0 (44.9–47.1)	28.8 (27.6–30.0)
pT3	13.9 (13.2–14.7)	21.6 (20.5–22.8)
pT4	7.2 (6.6–7.8)	3.0 (2.6–3.5)
pTx	10.2 (8.1–12.2)	16.9 (15.9–17.9)
Clinical *N* stage		
cN0	2.9 (2.6–3.1)	9.6 (2.8–22.1)
cN1	51.2 (50.3–52.0)	73.5 (58.2–88.8)
cN2	4.9 (4.4–5.4)	9.2 (2.2–16.1)
cN3	0.8 (0.6–1.0)	6.6 (6–7.3)
Pathologic *N* stage		
pN0	36.2 (35.4–36.9)	13.5 (12.5–14.4)
pN1	16.6 (15.8–17.4)	29.3 (28.1–30.6)
pN2	9.6 (9.0–10.3)	23.9 (22.7–25.1)
pN3	4.7 (4.3–5.2)	12.0 (11.2–12.9)
pNx	0.1 (0.1–0.2)	12.9 (12.0–13.8)
Grade		
GI	12.9 (12.2–13.6)	8.8 (8.4–9.2)
G2	54.4 (53.3–55.5)	45.9 (45.2–46.6)
G3	15.0 (14.3–15.8)	31.5 (30.8–32.1)
Gx	5.6 (5.1–6.1)	12.1 (0.9–25.1)
Mean duration of endocrine therapy (weeks)	23.14	—
Breast surgery		
Lumpectomy	45.0 (43.8–46.1)	26.9 (26.3–27.5)
Mastectomy	54.9 (53.8–56.1)	73.1 (72.5–73.7)
Axillary surgery		
Sentinel lymph node biopsy (SLNB)	46.1 (44.9–47.4)	34.1 (33.3–34.9)
Axillary lymph node dissection (ALND)	39.1 (37.9–40.3)	58.5 (57.7–59.3)
pCR		
Breast pCR	2.2 (0.2–4.2)	6.7 (6.3–7.0)
Node pCR	8.9 (8.0–9.8)	14.9 (9.8–18.7)
Complete pCR	2.3 (1.3–3.3)	4.9 (2.2–7.5)

IDC: invasive ductal carcinoma, ILC: invasive lobular carcinoma, pCR: pathologic complete response.

**Table 3:** Comparing the node pCR between NAC and NET in HR+, HER2−, cN+ patients.

*N*	Author	Definition of cN+	NET cN+ *N*	Node pCR by NET *N* (%)	NAC cN+ N	Node pCR by NAC *N* (%)	*P* value
1	Fontein [28]	Cytologically confirmed	25	3 (12)	—	—	—
2	Wright [33]	FNA, cytologically confirmed	40	0 (0)	58	4 (6.9)	0.091
3	Thornton [32]	Presence of features highly suspicious for malignancy on imaging or biopsy proven (NCDB)	855	69 (8.1)	5087	684 (13.4)	<0.001
4	Montagna [30]	Radiologic suspicious, biopsy proven in 83%	46	5 (11)	338	30 (8.9)	0.37
5	Hammond [25]	Presence of features highly suspicious for malignancy on imaging or biopsy proven	39	1 (2.6)	—	—	—
6	Kim [27]	Histologically proven by FNA or CNB	87	4 (4.6)	87	12 (13.8)	0.036
7	Kantor [29] DFBWCC	Histologically proven by CNB	23	1 (4.3)	—	—	—
8	Reis [23]	Radiologic suspicious (US or MRI), biopsy proven in 69.7%	33	6 (24.2)	—	—	—
9	Schipper [24]	Histologically proven by FNA or CNB	561	41 (7.3)	—	—	—
10	Cao [31]	Presence of features highly suspicious for malignancy on imaging or biopsy proven (NCDB)	1915	231 (13.5)	8673	1760 (20.3)	<0.001
11	Gwark [26]	Histologically proven by FNA or CNB	83	4 (4.8)	87	12 (13.8)	0.035
Total			3,707	Pooled estimate proportion % (with 95% CI)	14,330	Pooled estimate proportion % (with 95% CI)	
				8.9 (8.0–9.8)		14.9 (11.2–18.7)	

## Data Availability

The data that support the findings of this study are available on request from the corresponding author. The data are not publicly available due to privacy or ethical restrictions.

## Additional Points

*Synopsis*. (i) Considering the lower toxicity of NET compared to NAC and the increased use of NET during the pandemic, we performed a systematic review and meta-analysis to evaluate the role of NET in axillary management in cN+, HR+, HER2− patients.
